# Noninferiority trials: a specific design with a particular methodology

**DOI:** 10.1186/s13613-022-01020-w

**Published:** 2022-05-25

**Authors:** Martin Ruste, Jean-Luc Fellahi, Matthias Jacquet-Lagrèze

**Affiliations:** 1 Service d’anesthésie-reanimation, Hôpital Louis Pradel, Hospices Civils de Lyon; 59, Boulevard Pinel, 69677 Bron Cedex, France; 2 Faculté de Médecine Lyon Est, Université Claude Bernard Lyon, 1, 8, Avenue Rockefeller, 69373 Lyon Cedex 08, France; 3 Laboratoire CarMeN, Inserm UMR 1060, Université Claude Bernard Lyon 1, Lyon, France

We read with a great interest the article by Franco et al. [1] aiming to compare a dobutamine-sparing strategy to a dobutamine-to-all strategy in cardiac surgery. The authors addressed a crucial question in a field where randomized evidence is needed. After a careful reading of the manuscript, we have some questions regarding the noninferiority design and its interpretation. Noninferiority trials are infrequent and their methodology are poorly known by researchers, reviewers and readers while some of their fundamental particularities must be reported using a strict methodological approach [2].

The noninferiority approach is justified by the authors arguing that a superiority trial would have required more patients. Statistically, sample size required in noninferiority trials depends on the a priori definition of the alpha risk, the power, the noninferiority margin (delta), and the estimated prevalence of the main outcome in the two groups. Indeed, assuming the interventional strategy will have a small beneficial effect, decreases drastically the required sample size [3]. Unfortunately, this last criterion is lacking, preventing the verification of the sample size calculation. Nevertheless, we calculated with the observed proportion of events in each group (*P* = 0.31, *P* = 0.34) the a posteriori power of the study to be 0.54, and the required sample size to demonstrate the noninferiority between groups to be 161 patients in each group, suggesting the study is markedly underpowered.

In a noninferiority trial, the null hypothesis (to be rejected) is that the treatment is inferior, whereas the alternative hypothesis (to be proven) is that the treatment is non-inferior. The type I and type II errors are hence reversed, if compared with superiority trials. To demonstrate the noninferiority of the dobutamine-sparing *vs.* dobutamine-to-all strategy considering mortality and major cardiovascular events (i.e*.*, a failure criteria where lower is better), the upper limit of the difference between confidence intervals has to be lower than the noninferiority margin [3]. Moreover, the *P*-value calculated for noninferiority has to be inferior to the alpha risk to reject the null hypothesis. It is a different *P*-value from a non-significant *P*-value for superiority. In other words, “the absence of evidence does not mean the evidence of absence” [2]. Here, the confidence interval of the difference between both groups contains the noninferiority margin (absolute difference between groups: 2.5% (95% CI −11.8 to 16.7), noninferiority margin 10%) and the *P*-value, for which it was not determined if the test was a superiority or a noninferiority test, is higher than statistical significance (*P* = 0.74). Noninferiority was subsequently non-established (probably because the study was underpowered) and we disagree with the authors’ conclusion that dobutamine-sparing is non-inferior to dobutamine-to-all strategy. To limit this confusion, as proposed in the extension of the CONSORT statement 2010 for reporting of noninferiority and equivalence randomized trials CONSORT considering noninferiority trial [4], a simple figure illustrating the difference between groups with its confidence interval and the noninferiority margin would emphasize this issue (Fig. 1).

**Fig. 1:**
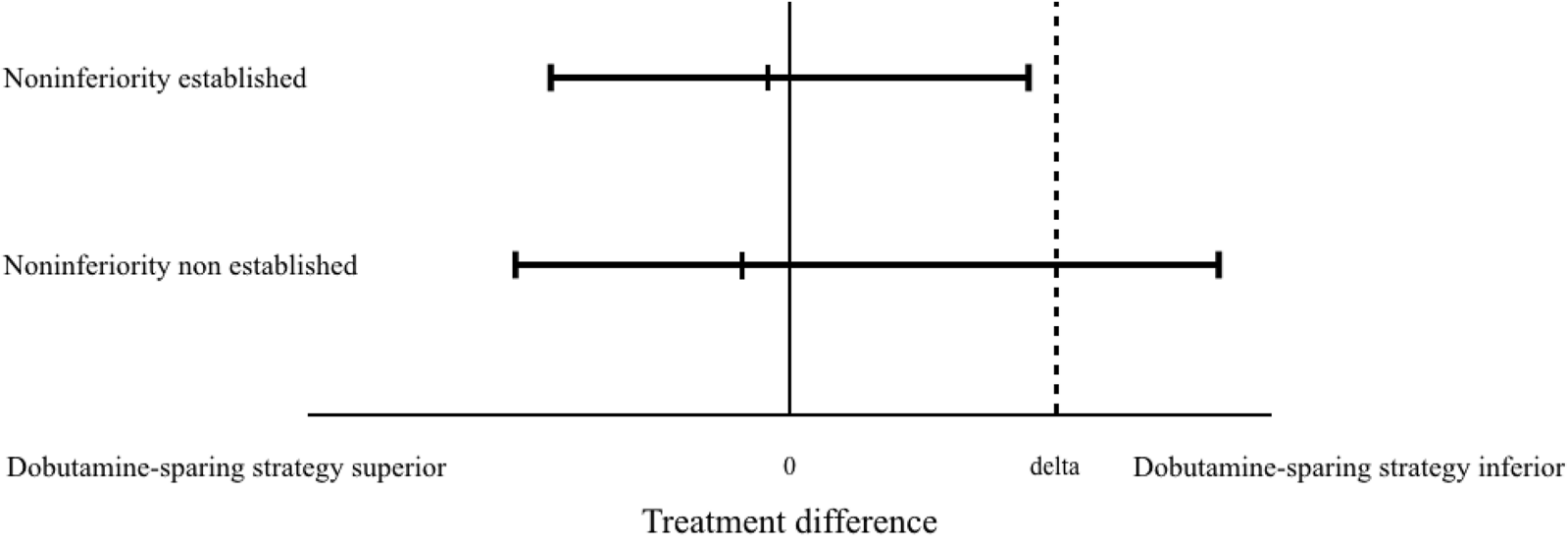
Graphical representation in noninferiority trial if efficacy is measured by failure rates (lower is better). Delta: margin of noninferiority

To conclude, noninferiority (or equivalence) trials are infrequent in the critical care scientific literature and required specific statistical hypotheses that must be well understood by researchers and readers. As previously reported [5], this noninferiority trial is at risk of misinterpretation and misleading conclusions.

## Data Availability

Not applicable.
